# Objective sleep quality and MRI biomarkers of Alzheimer’s Disease in a diverse cohort of older adults

**DOI:** 10.1002/alz70862_110166

**Published:** 2025-12-23

**Authors:** Dillys Xiaodi Liu, Clémence Cavaillès, Meredith N. Braskie, Yue Leng, Katie L Stone, Kristine Yaffe

**Affiliations:** ^1^ Department of Psychiatry and Behavioral Sciences, University of California, San Francisco, San Francisco, CA USA; ^2^ Imaging Genetics Center, Mark and Mary Stevens Neuroimaging and Informatics Institute, Keck School of Medicine, University of Southern California, Marina del Rey, CA USA; ^3^ University of California, San Francisco, San Francisco, CA USA; ^4^ California Pacific Medical Center Research Institute, San Francisco, CA USA; ^5^ Department of Psychiatry, Neurology, and Epidemiology and Biostatistics University of California San Francisco School of Medicine, San Francisco, CA USA

## Abstract

**Background:**

Poor sleep is linked to higher risk of Alzheimer’s Disease (AD), yet its relationship with AD‐related biomarkers is not fully understood. This study aimed to examine associations between objective sleep characteristics and three AD‐related magnetic resonance imaging (MRI) biomarkers in a diverse cohort of community‐dwelling older adults.

**Method:**

The Dormir Study recruited participants aged 50 and older enrolled in the ongoing Health and Aging Brain Study‐Health Disparities (HABS‐HD) cohort. Sleep characteristics were objectively measured using 7‐day wrist actigraphy, capturing nighttime sleep duration, total time in bed (TIB), sleep efficiency (SE), wake after sleep onset (WASO) time, average awakening length, and sleep fragmentation index. AD‐related MRI biomarkers included mean hippocampal volume, AD‐signature cortical thickness, and white matter hyperintensity (WMH) volume. We performed multivariable linear regressions adjusting for age, sex, race/ethnicity, education, cognitive status, body mass index, smoking, alcohol consumption, and MRI scanners. We reported results using coefficient per 1‐standard deviation increase in the continuous sleep exposures.

**Result:**

We examined 703 participants (mean age 66.4±8.4 years, 65.6% women), including 164 (23.3%) Black, 223 (31.7%) Hispanic, and 316 (45.0%) Non‐Hispanic White individuals. Seventy‐nine (11%) had nighttime sleep duration<6 hours, 462 (66%) had 6‐8 hours, and 162 (23%) had >8 hours. After multivariable adjustment, nighttime sleep duration>8 hours [*β* = ‐0.16, 95% confidence interval (CI) ‐0.32, ‐0.002; compared to participants who slept 6‐8 hours], longer TIB (*β* = ‐0.12, 95%CI ‐0.19, ‐0.05), WASO (*β* = ‐0.08, 95%CI ‐0.15, ‐0.01), and awakening length (*β* = ‐0.10, 95%CI ‐0.17, ‐0.03) were associated with smaller hippocampal volume. Similar patterns were observed for nighttime sleep duration>8 hours (*β* = ‐0.23, 95%CI ‐0.41, ‐0.04), longer TIB (*β* = ‐0.09, 95% CI ‐0.17, ‐0.02) and awakening length (*β* = ‐0.12, 95% CI ‐0.20, ‐0.04) with thinner cortex in AD‐signature regions, but not for other sleep characteristics. No associations were found between sleep characteristics with WMH volume.

**Conclusion:**

Nighttime sleep duration>8 hours and poor sleep quality, including longer TIB, WASO, and awakening length, were associated with worse AD‐related MRI patterns among diverse community‐dwelling older adults. Future longitudinal studies are warranted to validate the pathophysiological mechanisms underlying those sleep‐AD associations.

